# Assessment of Diet and Physical Activity of Brazilian Schoolchildren: Usability Testing of a Web-Based Questionnaire

**DOI:** 10.2196/resprot.2646

**Published:** 2013-08-19

**Authors:** Filipe Ferreira da Costa, Camilie Pacheco Schmoelz, Vanessa Fernandes Davies, Patrícia Faria Di Pietro, Emil Kupek, Maria Alice Altenburg de Assis

**Affiliations:** ^1^Graduate Course in Physical EducationDepartment of Physical EducationFederal University of Santa CatarinaFlorianopolisBrazil; ^2^Graduate Course in Public HealthDepartment of Public HealthFederal University of Santa CatarinaFlorianopolisBrazil; ^3^Graduate Course in NutritionDepartment of NutritionFederal University of Santa CatarinaFlorianopolisBrazil

**Keywords:** usability testing, questionnaire, physical activity, diet, children

## Abstract

**Background:**

Information and communication technology (ICT) has been used with increasing frequency for the assessment of diet and physical activity in health surveys. A number of Web-based questionnaires have been developed for children and adolescents. However, their usability characteristics have scarcely been reported, despite their potential importance for improving the feasibility and validity of ICT-based methods.

**Objective:**

The objective of this study was to describe the usability evaluation of the Consumo Alimentar e Atividade Física de Escolares (CAAFE) questionnaire (Food Consumption and Physical Activity Questionnaire for schoolchildren), a new Web-based survey tool for the self-assessment of diet and physical activity by schoolchildren.

**Methods:**

A total of 114 schoolchildren aged 6 to 12 years took part in questionnaire usability testing carried out in computer classrooms at five elementary schools in the city of Florianopolis, Brazil. Schoolchildren used a personal computer (PC) equipped with software for recording what is on the computer screen and the children’s speech during usability testing. Quantitative and qualitative analyses took into account objective usability metrics such as error counts and time to complete a task. Data on the main difficulties in accomplishing the task and the level of satisfaction expressed by the children were assessed by the observers using a standardized form and interviews with the children. Descriptive statistics and content analysis were used to summarize both the quantitative and the qualitative aspects of the data obtained.

**Results:**

The mean time for completing the questionnaire was 13.7 minutes (SD 3.68). Compared to the children in 2nd or 3rd grades, those in 4th or 5th grades spent less time completing the questionnaire (median 12.4 vs 13.3 minutes, *P*=.022), asked for help less frequently (median 0 vs 1.0 count, *P*=.005), had a lower error count (median 2.0 vs 8.0 count, *P*<.001), and obtained a higher overall performance score (median 73.0 vs 68.0, *P*=.005). Children with a PC at home spent less time completing the questionnaire (median 12.3 vs 14.9 minutes, *P*<.001), had a lower overall error count (median 2.0 vs 9.0 count, *P*=.03), and had a higher performance score (median 72.0 vs 64.0, *P*=.005) compared to the children without a PC at home. The most common difficulty in completing the questionnaire was in using the scroll bar. The majority of children reported a positive evaluation (liked a lot or liked) for the four design elements, which were evaluated.

**Conclusions:**

The results of the present study provided feedback to improve the final version of the CAAFE questionnaire. Quantitative data showed minor errors and system failures, while qualitative data indicated that, overall, the children enjoyed the CAAFE questionnaire. Grade levels and PC use must be taken into account in Web-based tools designed for children.

## Introduction

Obesity is a major public health problem worldwide, affecting people of all ages. In past decades, developed countries have experienced a rapid growth in childhood obesity, as have developing countries such as Brazil [1]. According to a survey performed on 9,018 Brazilian children in 2008-2009, one-third of them (~3006 children) aged 5 to 9 years were found to be overweight and 1497 (16.60%) were obese. These figures are worrying compared to those of 1970s, when less than 10% of the children were overweight and 3% were obese [2]. Although obesity epidemics have complex causes, physical activity and diet are the two main determinants of energy balance, and are therefore directly related to the worldwide increase in obesity. To tackle the pediatric obesity epidemic, monitoring tools for nutritional status, physical activity, and diet can be very helpful in determining the effectiveness of policies and health promotion programs.

Due to their importance and strategic role in promoting the health of young people, several survey systems have been specially designed to monitor the physical activity, diet, and nutritional status of schoolchildren [3-5]. All these systems rely on self-reported assessment, as this is the most practical and cost-effective way of collecting data for survey purposes. However, children’s self-reported assessments are often prone to bias, as they rely on their cognitive ability to recall details of both physical activities (eg, type, frequency, duration, intensity) [6] and dietary habits (eg, the types and amount of food consumed) [7]. Therefore, the data collection instruments designed for children must be adapted in terms of language and cognitive demand to provide reliable and valid data.

In 2002 and 2007, two surveys using similar methods to investigate nutritional status in schoolchildren and its association with behavioral and sociodemographic factors were conducted in the city of Florianopolis in Southern Brazil [8-10]. A paper-and-pencil questionnaire was used to assess the food consumption and physical activity of schoolchildren aged 7 to 10 years [11]. The validated paper-and-pencil version of the questionnaire titled “Previous Day Food Questionnaire (PDFQ)” was subjected to reliability and validity studies. The results of the reliability study indicated that agreement level between answers from the questionnaire and the observations of school meals was moderate or high for all food categories [11]. In the validity study of the third version of the PDFQ, mean values for sensitivity and specificity were 70.5% and 87.1%, respectively, for 12 food items in three combined school meals [12]. Based on this experience, we developed the CAAFE questionnaire: Consumo Alimentar e Atividade Física de Escolares (Schoolchildren Food Consumption and Physical Activity), a new tool for monitoring the physical activity and diet of young children in Web-based surveys.

The access of Brazilian students to new information and communication technologies (ICTs) has increased as a result of national policies to equip public schools with computers and Internet (eg, Programa Nacional de Tecnologia Educacional—ProInfo and Programa Um Computador por Aluno) [13]. In Florianopolis, the city where the software CAAFE has been tested, all public and private schools are equipped with computer labs and Internet. Taking into account the infrastructure presented in the school setting, the increasing familiarity of children with ICT, and the scarcity of data on the diet and physical activity of Brazilian schoolchildren, it is expected that an online self-reported assessment like the CAAFE questionnaire will be a feasible and cost-effective tool capable of providing data for the planning of health policies.

Web-based health surveys are a promising and attractive alternative to traditional data collection methods. They have several advantages over paper-and-pencil surveys such as standardized questionnaire administration, the elimination of interviewer-associated bias, reduced survey costs, and improved data quality due to automated data checking and storage [14,15]. The past decade has seen the rapid development of several ICT-based tools for assessing diet and physical activity [16-19]. A number of these tools were developed specifically for young people and seem to be feasible for survey purposes [18-29]. Despite their advantages, Web- and technology-based tools are prone to producing measurement bias in addition to that produced by traditional methods. Simple errors such as not scrolling down to see an entire webpage or difficulties in understanding how to correct an erroneous response may lead to invalid data [30]. Some of these errors may occur as a result of poor usability [31].

As far as we are aware, there are few Web-based physical activity and food consumption assessment tools that have presented the results of usability testing [32,33], particularly among schoolchildren [32]. Since usability is an instrument attribute of utmost importance in providing more reliable and valid data, a detailed description of usability testing can promote its use for the development of tools, and provide more quantitative and qualitative data for study comparisons. To that end, usability evaluation of the CAAFE questionnaire is the first step in establishing its feasibility as a tool for Web-based health surveys. The aim of this paper is to describe the general attributes of the CAAFE questionnaire as well as to give a detailed description of its usability characteristics.

## Methods

### Description of the CAAFE Questionnaire

The CAAFE questionnaire was programmed according to international quality standards (CMMI level II) in Brazil by a Web-based system/mobile application company. The questionnaire is a cross-platform (Win/Linux/Mac OS) and browser-based software written using PHP5, HTML5, CSS3, and JavaScript. Two-dimensional images integrated into a larger scene (sprites) were used, along with CSS programming and JavaScript language management to animate an avatar that helps children to complete the questionnaire. The application runs successfully on browsers such as Internet Explorer, Firefox, Chrome, and Safari. It requires Internet access and speakers (or headphones), and displays best with the current standard screen resolution (1024 × 768 pixels).

The CAAFE questionnaire was developed for school-based surveys of 7 to 10-year-old children. Schoolchildren can access the CAAFE questionnaire by logging on with a password automatically created by the system and specific for each survey, school, and time of day (morning or afternoon), to prevent the same respondent from answering the questionnaire more than once in a day.

In the formative research to develop the CAAFE questionnaire, several data sources were taken into account including: (1) our experience with the validation studies conducted with the paper-and-pencil versions of the PDFQ [11,12] and Previous Day Physical Activity Questionnaire (PDPAQ) [34], (2) food consumption and physical activity data reported by schoolchildren participating in school surveys using the PDFQ and PDPAQ [9,10], (3) previous Web-based instruments designed for schoolchildren [18-20,22-24,35], (4) data from focus groups conducted with physical education (PE) teachers of schoolchildren [36] and nutritionists working with the school meals program, (5) 7-day food and activity diaries completed by 180 schoolchildren from three schools, and (6) research team discussions with a specialist in child psychology and education to choose the questions and the Web layout according to the children’s cognitive abilities. The main lessons learned in the formative research process indicated that the instrument must: (1) be as simple as possible both in the quantity and quality of information required from the children, (2) be attractive and interactive, and (3) include only relevant items to be marked by the children. A more complete description of the formative process of the CAAFE questionnaire will be provided in an upcoming publication.

The CAAFE questionnaire is a single-day recall procedure divided into three sections: (1) registration form, (2) diet, and (3) physical activity. The registration section refers to information about respondents, such as their name, mother’s name, sex, weight, height, age, date of birth, and study period. These are simple questions with each screen presenting only one question. The option “I don’t know” is offered for almost all the questions, to avoid frustration when the respondent is unsure about the answer. Four questions require the answers to be typed (ie, the name, mother’s name, weight and height of the respondent), whereas all the other questions could be answered only by clicking on large icons. In the CAAFE survey system, it is planned that all participants will have their weight and height measured 2 weeks before completing the questionnaire, and will keep these measurements to assist them in answering the survey. The name of the school, city, and state are automatically entered on the database, based on a previously compiled list linked to the password entered. In case of response interruption (eg, Internet disconnected), children can continue the questionnaire at a later time on the same day. Otherwise, children would have to start the questionnaire all over again.

**Figure 1:**
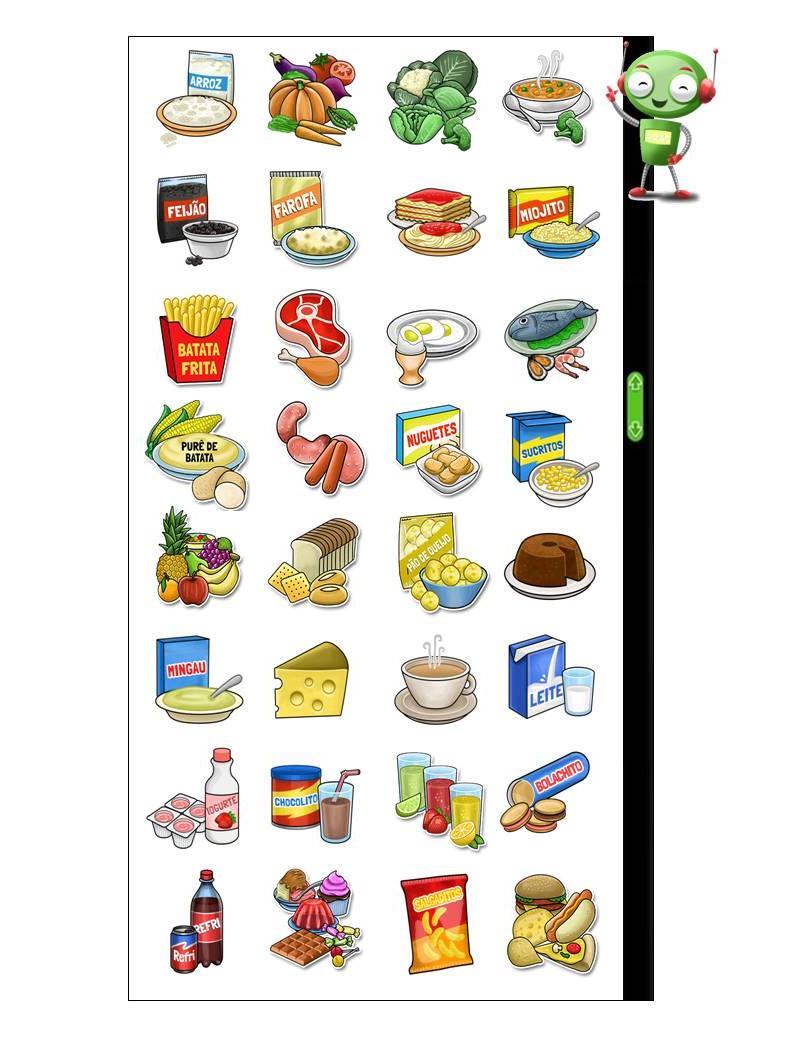
The CAAFE questionnaire food options.

The food consumption section of the questionnaire was divided into six parts (breakfast, morning snack, lunch, afternoon snack, dinner, and evening snack), to help children recall foods and beverages consumed during the previous day. This approach has been frequently used in questionnaires for young children, as it facilitates the recall process [11,18,19]. The foods and food groups, including healthy and unhealthy items, were chosen taking into account the food patterns of children in this age group (reported by schoolchildren in the 7-day food diaries), foods offered in school meals, and foods recommended in the Brazilian Food Guidelines. Upon choosing the food items from a screen with 32 food icons (see Figure 1), schoolchildren can check all six meals to add or exclude specific items. After completing the six-meal events, four questions are asked on different screens: (1) “Did you have a school meal yesterday?” (yes/no); (2) If yes, “Which of these foods did you eat during the school meal?”; (3) “How many times per week do you have school meals?” (none, 1, 2, 3, 4, every school day); and (4) “How do you like the food served in the school meal?” (five options illustrated by a hedonic scale). The meals listed in question 2 are those likely to be offered in the school setting (the first four meals of the day), and include only the foods/beverages previously marked by the children.

**Figure 2:**
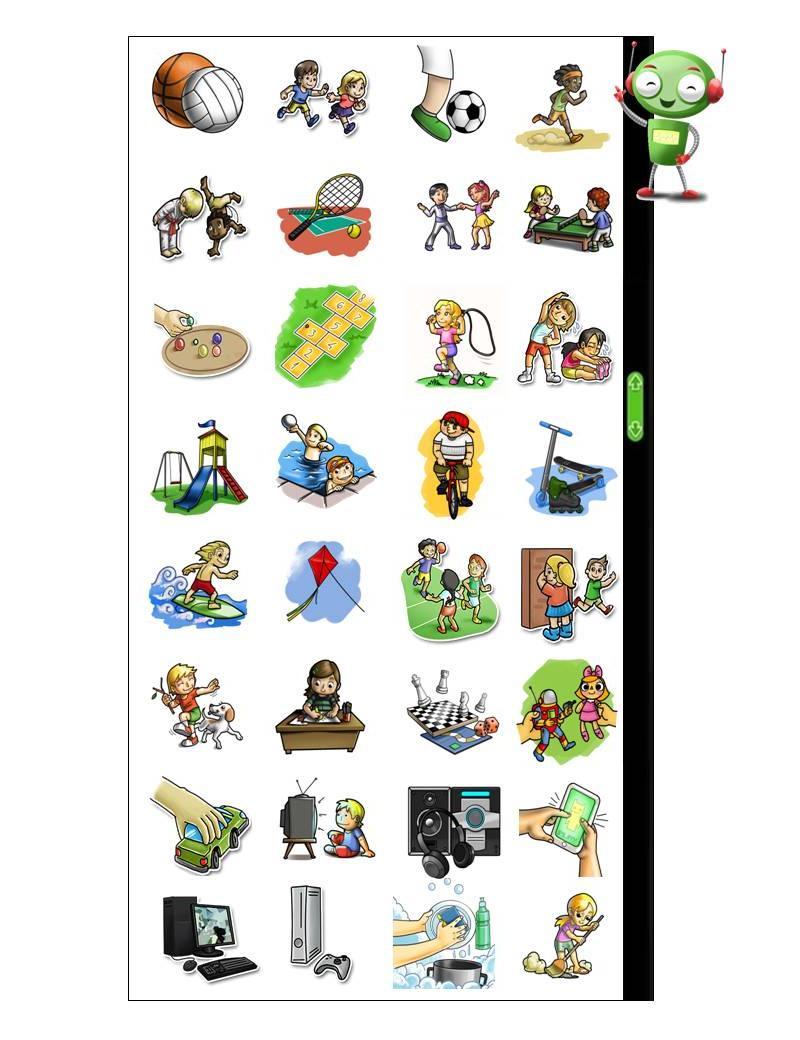
The CAAFE questionnaire activity options.

The physical activity section is divided into the three parts of the day (morning, afternoon, and evening). Other ICT-based questionnaires have used this strategy to help children recall the physical activities performed [22,23,35]. A closed list of leisure activities, sports, home chores, and sedentary activities was compiled based on the results from focal groups, previous instruments for this age range, and the 7-day recall completed by 180 schoolchildren. Children can choose sedentary and physical activity icons from among 32 options (see Figure 2). When a physical activity is chosen, a modal window opens and the avatar asks the children about its intensity, “How tired did you get during this activity?” and three illustrated icons depicting different degrees of physical exertion are presented. As in the food section, children can check their answers and make changes if necessary in this section also. Next, the avatar asks five questions on separate screens: (1) “Click on the activities you did yesterday when the teacher was present.”; (2) “Did you have a PE class yesterday?” (yes/no); (3) “How many days per week do you have PE classes?” (none, 1, 2, 3, 4, every day of the week); (4) “How do you like PE classes?” (five options on a hedonic scale); and (5) finally, children are asked about the mode of transport they use to travel to school (car, school bus, regular bus, motorcycle, on foot, bike, skateboard, or boat).

**Figure 3:**
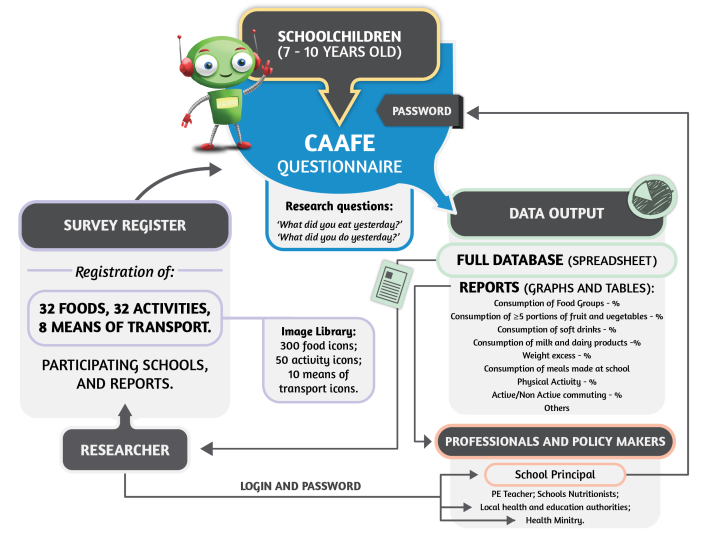
Functionality of the CAAFE survey system: survey register, children's responses, and data output.

The final CAAFE questionnaire flow can be accessed in Multimedia Appendix 1. The total number of screens can vary according to the responses chosen (eg, omitting questions regarding the previous school day if the child did not go to school that day). Figure 3 presents the major functionality of the CAAFE survey system as well as the interaction between researchers, children, professionals, and policy makers.

### Usability Testing

#### Overview

The most basic and useful method for improving the website usability is user testing that contains three components: (1) selecting representative users, (2) asking them to perform representative tasks with the Web-based interface, and (3) observing where they succeed and where they have difficulties with the user interface [31]. Several methodologies are available for usability testing, depending on the study objectives and on limitations such as time, money, management backing, development team support, and ability to recruit participants [37]. In the present study, we opted to adapt the multiple testing product version method described by Rubin and Chisnell [37]. Our strategy differs from the original method in that each software version was tested by different children.

#### Pilot Test

A pilot test of usability for the first version of the CAAFE questionnaire was conducted with 110 children (85% of the eligible subjects from five selected classes) aged 7 to 13 years (59 girls/110 children, 53.6%), from two elementary public schools in the city of Florianopolis, the capital of the Santa Catarina, Brazil. This first version had a different questionnaire flow compared to the final version previously described. The metaphor of a diary was used to help children record what they ate/did over the course of the previous day. Both physical activities and food consumption were assessed in the same screen.

The same usability test procedures were used for the pilot test. Several design elements caused disruptions in the flow (eg, hidden buttons, poor quality of drawings), and the children were unable to complete the questionnaire without assistance. In addition, almost all the children (109/110, 99.1%) enjoyed answering the questionnaire, and only a few children reported they had any difficulty completing the task. The mean time to complete the first version was 29 minutes. The low usability performance of this version led to the redesign of the software layout. The main improvements to the final version of the software were the presentation of diet and physical activity sections in separate screens. Improvements were also made in the Web design.

#### Participants

Six other classes were invited to test the second version of the software. To represent schoolchildren from different socioeconomic backgrounds, four public schools from different regions of the city (central, north, east, and south) were selected. A total of 114 (114/152, 75.0% of the eligible subjects) schoolchildren aged 6 to 12 years (58 girls/114 children, 50.8%) took part in this phase. In order to assess child computer use, all parents answered a questionnaire on the number of computers at home (none, 1, 2, 3, 4, or more), child access to computers at home (yes, no, or sometimes), a computer in the child’s bedroom (yes/no), main use of the computer by the child (Internet search, games, social network sites, and/or schoolwork), frequency of computer use in a typical week (none to every day), and average time spent on the computer per day (less than one hour to more than five hours).

Both studies were approved by the Human Research Ethics Committee of the Universidade Federal de Santa Catarina (protocol 2250/11). All children gave their oral consent, and parents or guardians were asked to authorize their participation by signing an informed consent form.

#### Location, Equipment, and Schedule

The location of the test sessions is closely linked to the study design and users [37]. In the present study, all sessions were carried out in the computer labs of the participating schools. There were two main reasons for this: (1) children were familiar with the school computer labs because several academic and nonacademic assignments had been completed there, and (2) school computer labs will be the primary setting for the CAAFE survey system in the near future.

In order to record screen logs and user audio during usability sessions, team laptops were equipped with Morae Recorder. Along with Morae Manager, this software kept a record of the children’s navigation process through the CAAFE questionnaire, compiling these data for a subsequent review. A personal computer (PC) monitor, keyboard, and mouse were attached to the research team laptops (equipped with a Morae Recorder) once the children became familiar with the computers. Sessions were arranged in advance with school staff and carried out in November and December 2012.

#### Test Session Protocol

At the start of the session with children, the moderator introduced the task to the entire class. The children were told that we were testing software and would like to improve it with their help. Small groups of children (n=3-6) were taken to the computer lab according to observer availability. Upon arrival at the computer lab, the children were again introduced to the task and a few questions were asked in order to put them at ease (eg, What is your name? How old are you? Do you have a computer at home? What do you enjoy doing on your computer?). The next step was to introduce each child to one of the observers (one observer per child), who told them that an avatar would explain the task. Each child answered the questionnaire as independently as possible while the observer took notes about their performance. The observers were instructed to position themselves at a desk outside the child’s line of sight and to give minimal feedback when asked for help. They were also instructed to encourage the child to try again.

The observer protocol included a four-level performance scale (0, 1, 2, 3) for the 25 screens in the CAAFE questionnaire, providing a total score ranging from 0 to 75 (25 × 3=75). Children’s requests for help and any other relevant observations were recorded in the appropriate fields. The scoring on the performance scale is interpreted as follows. Performance scale for level 0 signifies that the child is unable to fill out the questionnaire and continue the Web flow alone. It also interprets that child does not know what has to be done. Level 1 on performance scale tells that child has difficulty with typing, navigates and chooses icons slowly and/or with many unnecessary clicks on the screen, and gives long periods of inactivity on the same screen. Level 2 states that child types efficiently but with a few errors. Child may find difficulty in navigating and selecting icons/items. Score 3 on performance level demonstrates that child types efficiently without errors, and can navigate and choose the options quickly and efficiently. Child showing score 3 on the scale is said to be self-assured.

The children’s primary task was to complete the entire questionnaire. Immediately after the task, the observers were instructed to record their observations according to four predefined categories: (1) usability issues: child’s difficulties in moving forward and answering the questionnaire due to the Web design; (2) audio and visual elements: any specific comments regarding the icons, screens, or avatar speech; (3) system errors: any programming failure displayed by the system or interrupted Internet access; and (4) child performance, comments on computer skills (eg, using the mouse and scroll bar, typing), cognitive and emotional aspects: understanding the speech, reading and writing levels, and the behavior exhibited (eg, calm, agitated, anxious).

After the children completed the CAAFE questionnaire, an interviewer asked them about their satisfaction with the task performed and what they liked or disliked about the questionnaire. We used a standard form created by the research team. A five-level hedonic pictorial scale was used to evaluate specific aspects of the software layout, such as colors/screens, food and activity icons, and avatars. In addition, children were asked to describe their difficulties during the task and to suggest possible changes for improving the software.

### Analysis

#### Quantitative Analysis

For quantitative analysis, all recorded screen logs were reviewed and analyzed by the first two authors. The primary task was divided into three parts (registration form, diet, and activities) to describe usability issues in specific sections of the CAAFE questionnaire. Time to accomplish the task (average and range) was set from the first click on the “Start” button.

Each recorded screen was carefully reviewed to divide the main usability issues into four broad categories: Web design usability issues, response inconsistencies, requests for help, and system or Internet failure. Information on the response to the question “Have you already done this task before?”, name and date of birth, the time of day the children attend school (morning, afternoon, or both), and the number of PE classes were checked using information from the school staff, and served as the basis for assessing response inconsistencies. Height and weight were not measured in the usability study; therefore, the validity of this information could not be checked. Instead, we looked for “unlikely” data on height (eg, 2.5 m) and weight (eg, 10 kg) and categorized this as “response inconsistencies.” Height and weight will be measured in the validation study; we will therefore be able to detect mistakes in data entered by the children and to compare the mistakes with the measures previously collected. All registered occurrences were graphically presented. The three main errors, namely Web design usability issues, response inconsistencies, and request for help, system bugs, and Internet failure were identified for types II, III, and IV, while type I presented only a single error, that is Web design usability issues.

Usability attributes were compared according to gender, grade (2nd/3rd vs 4th/5th), and access to a computer at home (yes/no). The Wilcoxon-Mann-Whitney test was used in these comparisons because the distributions were either positively (time to accomplish the task, help request, or mean error count) or negatively (total performance score) skewed. We considered differences to be significant at the *P*<.05 level, and used SPSS version 21 for statistical analysis.

#### Qualitative Analysis

Qualitative Analysis Was Based on Observers’ Notes Made After the Children Completed the Caafe Questionnaire and on the Children’s Interviews. for the Former, the Most Relevant Usability Issues Were Highlighted Based on Four Prespecified Topics: (1) Children’s Difficulties in the Navigation Process (eg, to Move Forward After Answering a Question), (2) Audio and Visual Elements That Would Cause Doubts, or Any Comments Made by the Children, (3) System Errors That Occurred During Completion, and (4) Difficulties and Behaviors Displayed by the Children. the Children’s Impressions of the Software Were Described Based on Content Analysis Along With Frequency Distribution of Their Evaluation of the Key Elements of Software Design.

## Results

### Participant Characteristics

The characteristics of the 114 schoolchildren who participated in the study are presented in Table 1. It was found that 3 out of 4 children had a computer at home, while 16 of the 114 students (14.0%) had no access to computers outside the school environment. The average time spent on the computer was almost 10 hours/week, ranging from no use to 49 hours/week of computer use. The most common use of computers by children, as reported by their parents, was schoolwork and social networking, followed by Internet searches. This result partially contrasted with the data reported by the children in the pretest interview, in which games were cited as the most common computer use, followed by social networks (data not shown).

### Quantitative Results

**Figure 4:**
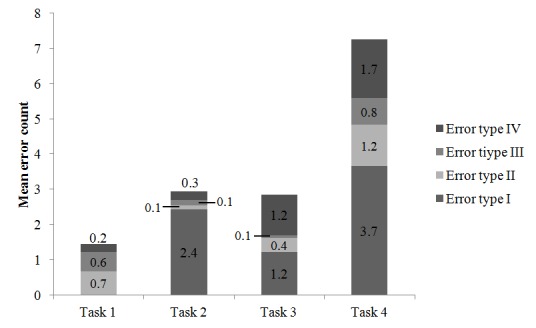
Mean error count by type according to usability test task. Error type I: Web design usability issues, Error type II: response inconsistencies, Error type III: requests for help, and Error type IV: system bugs and Internet failure.

The usability test sessions with 95 of the 114 participants were recorded, providing data for quantitative analysis (19 individual test sessions could not be recorded due to technical problems). The mean time to complete the registration form, diet, and activity sections was 3.5 min (SD 1.9), 5.7 min (SD 1.5), and 3.9 min (SD 1.1), respectively. The mean time to complete the entire questionnaire was 13.7 minutes (SD 3.7) (Task 4). Figure 4 shows the mean error count for each specific task. The mean error count for the entire questionnaire (Task 4) was 7.2 (median 4; range 0-22; interquartile range 11).

The most frequent difficulty encountered in the Task 1 was the request for help in typing in the respondent’s name (17/95, 18%). In addition, about 16 of the 95 children (17%) reported they had already answered the questionnaire when in fact they had not. In the diet and activity sections (Task 2 and 3), the most common error was related to Web design usability issues. In the physical activity section, 48% (46/95) of participants saw the “Continue” button before clicking in a response option. Moreover, about 36% (34/95) of children did not report the correct frequency of PE classes per week. Overall, the most common error in completing the questionnaire was difficulty in using the scroll bar (50/95, 52%). The top three error types are presented in Table 2.

Table 3 shows four usability performance indicators according to gender, grade, and the presence of a PC at home. No difference was found between genders. The children attending 4th and 5th grades spent less time completing the questionnaire, asked for help less frequently, had a lower error count, and had a higher overall performance score compared to those attending 2nd and 3rd grades. Children with a PC at home spent less time completing the questionnaire, had a lower overall error count, and obtained a higher performance score compared to those without a computer at home.

**Table 1:** Characteristics of study participants (N=114).

Characteristics		n (%) or mean (SD)
**Gender**		
	Boys	56 (49.1)
	Girls	58 (50.8)
Age (years)		9.2 (1.27)
**Grade**		
	2nd and 3rd	53 (46.4)
	4th and 5th	61 (53.5)
**Exposure to computer**		
	Computer at home (yes)	86 (75.4)
	No access to computer at home	28 (24.5)
**Access to a computer at home**		
	All the time	71 (62.2)
	Sometimes	27 (23.6)
	No access	16 (14.0)
Computer in the bedroom		26 (22.8)
Average time of computer use (hours/week)		9.8 (8.8)
**Computer use**		
	Internet search	43 (37.7)
	Games	38 (33.3)
	Social networking	54 (47.3)
	Schoolwork	54 (47.3)

**Table 2:** Top three errors by type in the usability test session.

Error type	Errors	Options
Error type I	Web design usability issues^a^	Poor scroll bar visibility in diet and activity sections
Error type II	Response inconsistencies	Number of physical education classes per week
		Children reporting they had already completed the task when in fact they had not
		Name misspelled
Error type III	Requests for help	To choose an option
		To type in own name
		To select date of birth
Error type IV	System bugs and Internet failure	“Continue” button visible before response options appeared on the screen
		Old version of the intensity scale for physical activities used in the questionnaire
		Need to click twice or more to continue the questionnaire

^a^There was only one error of type I.

**Table 3:** Usability performance according to gender, grade, and PC at home (N=95). All values are medians.

Usability attributes	Gender		*P* value^b^	Grade		*P* value^b^	PC at home		*P* value^b^
	Boys	Girls		2nd/3rd	4th/5th		Yes	No	
Time to complete (minutes)	12.9	12.8	.85	13.3	12.4	.030	12.3	14.9	<.001
Help request (count)	0.0	0.0	.50	1.0	0.0	.005	0.0	0.0	.174
Mean error count^a^	3.0	2.5	.93	8.0	2.0	<.001	2.0	9.0	.030
Performance score (0-75)	70.0	72.0	.63	68.0	73.0	<.001	72.0	64.0	.002

^a^Excluding system errors (type IV).

^b^ *P* values for the Wilcoxon-Mann-Whitney test.

### Qualitative Results

Table 4 shows the results obtained from the children’s interviews regarding satisfaction with the design elements of the CAAFE questionnaire. The majority of children reported a positive evaluation (liked a lot or liked) for the four design elements.

When children were asked about what they disliked about the CAAFE questionnaire, the majority reported liking everything. Two children disliked having to report their height. Two others considered the food section repetitive and the physical activity section boring. One child disliked the avatar’s voice, one complained about the sound volume, and another about the need to type in the full name.

When asked about the most difficult part of the CAAFE questionnaire, a majority of children answered that the task was easy to perform. The most common difficulty reported by the children was to recall what they had done the previous day (20/114, 17.5%). Some children (6/114, 5.2%) reported difficulty in remembering their height and weight. Less frequent difficulties cited by the children included typing their own name and that of their mother (4/114, 3.5%), indicating activities organized by an adult (1/114, 0.8%), finding a specific food item (n=1), and reporting the frequency of PE classes (1/114, 0.8%). Only a few children reported something they would like to change in the questionnaire. Some children (12/114, 10.5%) said that additional food/activity/transport options should be included. Some children (8/114, 7.0%) asked to include games related to food and physical activity.

**Table 4:** Frequency distribution (%) of the children’s qualitative evaluation of the design elements (N=114).

Satisfaction level	Design elements			
	Colors/Screens (%)	Food icons (%)	Activity icons (%)	Avatar (%)
Liked a lot	103 (90.0)	78 (68.4)	85 (74.6)	103 (90.3)
Liked	8 (7.3)	29 (25.4)	20 (17.5)	10 (8.7)
Neutral	3 (2.7)	4 (3.5)	9 (7.9)	1 (0.9)
Disliked	0 (0.0)	3 (2.6)	0 (0.0)	0 (0.0)
Disliked a lot	0 (0.0)	0 (0.0)	0 (0.0)	0 (0.0)

## Discussion

### Overview

The present study describes an interactive Web-based tool to assess the food consumption and physical activity of schoolchildren in a school-based survey, as well as the results of usability testing, which provided feedback and improved the final version of the CAAFE questionnaire. Quantitative data showed some minor errors and system failures, while qualitative data indicated that children enjoyed the CAAFE questionnaire. Subgroup comparisons demonstrated that children enrolled in higher grades and those who have a PC at home exhibited higher usability performance compared to children in lower grades and those who do not have a PC at home.

### Subgroup Usability Performance Comparisons

Markopoulos and Bekker [38] reported that several characteristics of children such as their capacity and inclination to verbalize, ability to concentrate, and motivation to perform the task may have an impact on usability testing. Gender, knowledge of language and general concepts, and prior experience with a PC are known to affect usability test results and were therefore included in the present study.

No gender differences were observed in usability performance. Gender differences in computer attitudes were reported in the early 1990s as a sex-role stereotyping issue [39], which would hypothetically lead to a gender effect on usability performance. However, recent studies have shown that gender differences are of little significance in this context [40,41]. Hypothetically, children born in the 21st Century have more experience with computers and other new information technology devices regardless of sex, which would explain similar performance in usability testing. These results partially differ from those of Ruggeri [32], who found that girls reported more difficulties in using the NUTRISIM, a new Web-based software for assessing diet of 10- to 14-year-old Brazilian schoolchildren.

As expected, children in higher grades exhibited better usability testing performance on the CAAFE questionnaire compared to those in lower grades. No other Web-based physical activity and/or diet assessment tool has reported such findings. This result is relevant to other Web-based questionnaires targeting a wider age range. The present study showed that children attending 2nd and 3rd grades needed more time to complete the task, asked for help more frequently, had a higher error count, and got a lower performance score. This indicates that special attention should be paid to younger children during data collection to minimize input data errors. However, it is interesting to note that our findings may not be generally applicable to all age groups. Younger children (11 year) demonstrated fewer difficulties in using the NUTRISIM compared to older children (12-14 year), although the latter reported fewer difficulties with regard to comprehension of the questions [32].

Another important factor in usability testing is computer knowledge and skills. Children who are more exposed to computers are expected to perform better in usability tests. In our study, having a computer at home was used as proxy of computer use. As expected, children with a PC at home showed improved usability performance compared to those without one. A preliminary question about computer ownership in the children’s families may help teachers and other staff members recognize difficulties in Web-based surveys designed for schoolchildren.

Regardless of differences in usability performance across grade levels and in PC ownership subgroups, the time to complete the task, requests for help, and error count were all acceptable, particularly considering the young age of the participants. First, the mean completion time of 13.6 minutes is deemed reasonable for children, as this is a sufficiently short time to hold their attention and to motivate them to complete the task. This time was even shorter than that reported to complete similar questionnaires for assessing physical activity among young children [18,22,23]. Second, the median of help requests was near to zero. Unfortunately, other questionnaires similar to ours have not provided the necessary information for comparison purposes of the mean error count and performance score.

### Software Improvements

The primary aim of usability testing is to identify and correct usability deficiencies [38]. Based on usability test results with end users, some improvements have been made in the CAAFE questionnaire. The biggest challenge was to make the scroll bar “visible” for children. Despite the fact that it is a common component in Web pages, 50 of the 95 (53%) children could not manage to use the scroll bar correctly. Some of them tried to click on the bar instead of dragging it down. A study with 7-year-old children who were inexperienced with a computer mouse supports this observation, since they were quicker and more accurate in pointing compared to dragging [42]. To solve the scroll bar problem in the CAAFE questionnaire, automated scrolling was set on each screen with a scroll bar. This allows children to see how the scroll bar works and how many food and activity options they can select.

In order to improve data quality from the question about the number of PE classes, we inserted the option “I don’t know.” Other minor changes were made without significantly altering the program. First, the “Continue” button was programmed to appear only after an answer was selected, thereby ensuring that children clicked on an option before proceeding to the next screen. Second, the intensity scale for physical activities illustrated in the first version was substituted by a new one.

The need to click two or more times to continue the questionnaire was probably related to the Internet speed, despite the fact that the software was designed and programmed to run with low-speed Internet connections. However, to minimize technical problems in future school-based surveys, the research team will provide minimal system and Internet access requirements to the computer labs of the participating schools, and will inform school principals about these requirements.

Error type II (response inconsistencies) and III (requests for help) were not solved by Web-design modifications. Instead, we expect to minimize these and other errors by way of appropriate training for the school staff where children will take part in the Web-based survey. In usability testing, the observers were trained to provide basic assistance to schoolchildren. However, school staff may be able to help children better with routine Web-based health surveys than outside observers. Tutorials and frequently asked questions on the CAAFE website will be made available to the school staff to standardize adult interventions during completion of the questionnaire.

### Strengths and Limitations

The present study exhibited a number of strengths that deserve to be mentioned. First, the participants and test setting closely mirrored the real context of future CAAFE Web-based surveys. Second, we gathered data using several methodological strategies (eg, direct observation, user interviews, test sessions recordings), which allowed us to obtain both quantitative and qualitative data for usability evaluation. Third, the number of participants was large enough to compare performance indicators across subgroups of interest. Nevertheless, some limitations must also be noted. The results should be interpreted with caution because methods and usability performance indicators were created especially for this study. Even though they are all natural extensions of existing performance indicators in various other contexts (eg, education, market research), direct comparisons with other studies in the field are limited. Furthermore, some children may have been unable to express their difficulties or to make appropriate suggestions for improving the software, or were too timid to do so during the testing sessions.

Accurate diet and physical activity assessment in young children is a challenge for researchers. Parents may help in reporting their children’ food intake in the home but often do not know what their consumption is outside the home. More precise methods such as 24-hour recalls and food diaries are costly and put too much burden on the respondents, making them impractical in a school setting. Self-report assessment of diet and physical activity among young children has a number of limitations such as social desirability, difficulties in recalling past events, and classifying and quantifying activities and food consumed. Children younger than 10 years are still developing cognitive abilities to accurately recall diet and physical activity [43,44], which must therefore be taken into account with any instrument designed for this age group. The CAAFE was designed considering the cognitive skills and literacy levels of children aged 7-10 years to respond to the questionnaire. The CAAFE was designed neither to estimate energy and nutrient intake, nor to estimate energy expense or to quantify activities performed. Questions about the size of food portions, or the frequency and duration of physical activities, were therefore not included. In addition, the closed list of foods and activities is not intended to represent the diversity of diet and activities. On the other hand, this simplifies the task of recall by prompting only the relevant food items eaten and the types of physical activities performed on the previous day, and also keeps the questionnaire relatively brief and easy both for completion and for administration. The cognitive task required for estimating portion size, frequency, and averaging may not be compatible with the perceptual and conceptual capacities of children who have not reached the stage of abstract reasoning, at approximately 10-11 years of age [43,44].

### Future Developments

The next step in the Web-based survey system is to provide data on the validity and reproducibility of the CAAFE questionnaire. Data collection is currently under way. It is expected that self-reported diet and physical activities will match the data that will be obtained by trained researchers through direct observation. Once the validity and reproducibility of the CAAFE questionnaire have been properly tested, a Web-based survey will be conducted in all 37 elementary public schools in the city of Florianopolis. The survey will provide data to evaluate response rate, technical and operational problems, as well as the time needed to complete the investigation. After further improvements have been made, a new Web-based survey will be conducted in other Brazilian cities to evaluate its feasibility as a national survey instrument. General information on the survey system is available on a CAAFE questionnaire website [45].

### Conclusions

The profusion of technology and information tools to assess physical activity and eating behavior in children has not been accompanied by adequate assessment of their usability features. This study sought to detail the methodological procedures and to report major usability problems found with end users of the CAAFE questionnaire, as well as to relate them to user characteristics. The results helped to improve the Web design and methodological procedures for future CAAFE Web-based surveys for schools. We expect the methods developed in this usability testing to be applied in other similar Web-based tools to compare and discuss results regarding their potential effects on instrument validity and reliability.

## Multimedia Appendix 1

CAAFE questionnaire flow.

## Abbreviations

CAAFE: Consumo Alimentar e Atividade Física de Escolares
ICT: information and communication technology
PC: personal computer
PDFQ: Previous Day Food Questionnaire
PDPAQ: Previous Day Physical Activity Questionnaire
PE: physical education
